# Deep Cerebellar Nuclei Play an Important Role in Two-Tone Discrimination on Delay Eyeblink Conditioning in C57BL/6 Mice

**DOI:** 10.1371/journal.pone.0059880

**Published:** 2013-03-26

**Authors:** Toshiro Sakamoto, Shogo Endo

**Affiliations:** 1 Laboratory for Behavioral Neuroendocrinology, Graduate school of Comprehensive Human Science, University of Tsukuba, Tsukuba, Ibaraki, Japan; 2 Department of Psychology, Faculty of Health Science, Kyoto Tachibana University, Yamashina, Kyoto, Japan; 3 Aging Regulation Research Team, Tokyo Metropolitan Institute of Gerontology, Itabashi, Tokyo, Japan; Centre national de la recherche scientifique, France

## Abstract

Previous studies have shown that deep cerebellar nuclei (DCN)-lesioned mice develop conditioned responses (CR) on delay eyeblink conditioning when a salient tone conditioned stimulus (CS) is used, which suggests that the cerebellum potentially plays a role in more complicated cognitive functions. In the present study, we examined the role of DCN in tone frequency discrimination in the delay eyeblink-conditioning paradigm. In the first experiment, DCN-lesioned and sham-operated mice were subjected to standard simple eyeblink conditioning under low-frequency tone CS (LCS: 1 kHz, 80 dB) or high-frequency tone CS (HCS: 10 kHz, 70 dB) conditions. DCN-lesioned mice developed CR in both CS conditions as well as sham-operated mice. In the second experiment, DCN-lesioned and sham-operated mice were subjected to two-tone discrimination tasks, with LCS+ (or HCS+) paired with unconditioned stimulus (US), and HCS− (or LCS−) without US. CR% in sham-operated mice increased in LCS+ (or HCS+) trials, regardless of tone frequency of CS, but not in HCS− (or LCS−) trials. The results indicate that sham-operated mice can discriminate between LCS+ and HCS− (or HCS+ and LCS−). In contrast, DCN-lesioned mice showed high CR% in not only LCS+ (or HCS+) trials but also HCS− (or LCS−) trials. The results indicate that DCN lesions impair the discrimination between tone frequency in eyeblink conditioning. Our results suggest that the cerebellum plays a pivotal role in the discrimination of tone frequency.

## Introduction

Eyeblink conditioning is one form of associative learning, which develops by the paired presentation of a neutral conditioned stimulus (CS), such as tones, and an unconditioned stimulus (US), such as electric shocks and air puffs. In delay conditioning the CS and US overlap temporally, although CS onset precedes US onset. The neuronal circuit for delay eyeblink conditioning has been extensively examined in rabbits, and the cerebellum is critical for conditioning in many species [1]–[8].

Recent developments and the availability of a variety of genetically-modified mice have made it possible to examine the role of certain neuronal cells and target molecules in the cerebellum that are specifically involved in eyeblink conditioning. However, genetically modified mice with altered cerebellar functions constantly result in the incomplete loss of conditioned responses (CR) during delay eyeblink conditioning [9]–[14]. Furthermore, lesions or inactivation of deep cerebellar nuclei (DCN) did not impair the acquisition and expression of delay eyeblink conditioning [12], [15]. We revealed that mouse DCN play important roles in delay eyeblink conditioning with the low intensity CS [16], but not with high intensity CS [15]. Furthermore, we revealed the essential role of the lateral amygdala for mouse eyeblink conditioning in response to high intensity CS [15]. Our findings suggest that the cerebellum is not involved in the acquisition of eyeblink conditioning when the high intensity CS is used in combination with the US of an electric shock.

Recently, the cerebellum was reported to be critical for not only motor coordination but also higher cognitive functions in mice, rats, and humans [17]–[21]. The cerebellum contributes to place learning, procedural learning and right/left discrimination in a maze, as indicated by the inferior performance of a cerebellar mutant or cerebellum-lesioned animals [19], [20], [22]–[25]. Therefore, we speculated that the cerebellum may be involved in more complicated eyeblink-conditioning paradigms when a high intensity tone is used as a CS.

In the present study, we focused on the discrimination of tone frequency in the eyeblink-conditioning paradigm and examined the role of DCN in this particular paradigm. DCN-lesioned and sham-operated mice were subjected to two-tone discrimination tasks: 1) the low-frequency tone CS (LCS: 1 kHz, 80 dB) and 2) the high-frequency tone CS (HCS: 10 kHz, 70 dB). In the two-tone discrimination task, a mouse is demanded to make CR with one CS (the CS+) that is paired with US, but not with different CS (the CS–) that is presented without the US. This task has two aspects, the acquisition of CR to one CS and the inhibition of CR to the other CS. Therefore, two-tone discrimination is more complicated associative learning than simple eyeblink conditioning. Our results indicate that the cerebellum is essential for the acquisition of more complicated tasks in eyeblink-conditioning paradigms, such as two-tone discrimination tasks.

## Materials and Methods

### Ethics Statement

Animal experiment protocols were approved and performed in accordance with the regulations outlined by the Japanese law and with the guidelines of Animal Use and Care Committee in Tsukuba University and Tokyo Metropolitan Institute of Gerontology.

### Animals

Male C57BL/6J mice aged 8–9 weeks old were used in all experiments. They were bought from a commercial breeder (CLEA Japan, Tokyo, Japan) 1 week before experimentation. Mice were kept under standard housing conditions with a 12-h light/dark cycle (lights on at 7∶00 A.M.) as previously described [15], [16]. All behavioral experiments were carried out during the light-on phase (8∶00 A.M. –5∶00 P.M.). Behavioral experiments were conducted in a blind manner.

### Surgeries

Mice were housed individually 1 week before surgery. Animals were anesthetized with a cocktail of ketamine (80 mg/kg) and xylazine (20 mg/kg) and fixed to a stereotaxic apparatus. Each mouse received surgery for electrolytic lesions (for details see, [15]).

#### DCN lesions

Bilateral electrical lesions of the DCN were made by a lesion-making device (Model 53500; Ugo Basile, Comerio, VA, Italy). Stereotaxic coordinates for lesions were based on the atlas of the mouse brain [26] as follows: AP = −6.0 mm from bregma; ML = ±0.8 and ±1.8 mm from midline; DV = −3.5 mm from skull. The electrode was inserted and lesions were made by passing a current at 0.75 mA for 10 s. In sham-operated mice, the electrode was inserted into the same position, but electrical current was not applied.

#### Electrode implanting for eyeblink recording

Mice were implanted with four electrodes (No.7910, A-M Systems, Carlsborg, WA, USA) attached to the left upper eyelid: Two were used to record the electromyogram (EMG) from the orbicularis oculi and to deliver electric shocks to periorbital tissues. Four wires were soldered onto a four-pin connector, which was attached to the skull using dental cement. Eyeblink responses were recorded with an EMG instrument [15], [16]. After surgery, DCN-lesioned mice were allowed to recover for 6–8 days, after which mice were subjected to simple eyeblink conditioning (Experiment 1) or rotor rod tests and two-tone discrimination tasks (Experiment 2).

### Eyeblink Conditioning

An eyeblink-conditioning system (EB-M4) was obtained from O’Hara and Co., Tokyo, Japan, and the conditioning was conducted as reported previously [15], [16]. Eyeblink conditioning was conducted in a cylindrical Plexiglas container placed in a sound- and light-attenuating box. A standard delay eyeblink-conditioning paradigm was used [15], [16].

**Figure 1:**
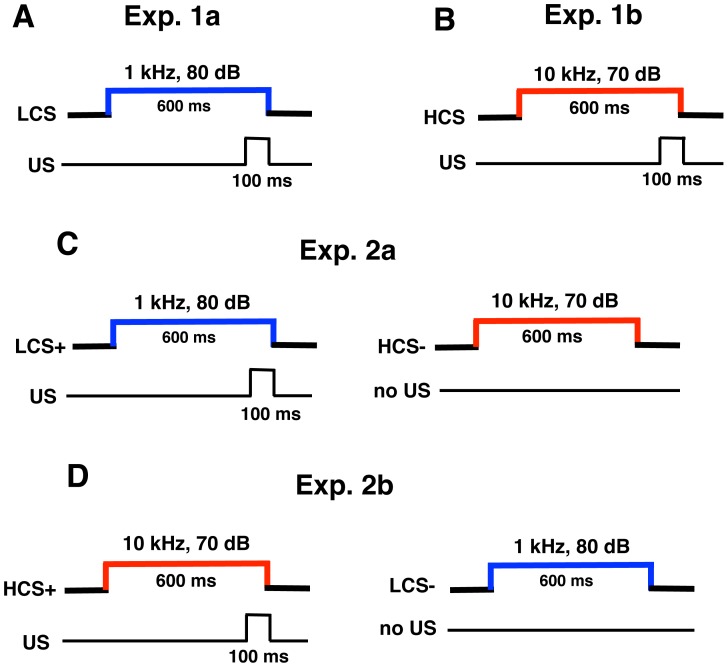
Temporal relationship of CS and US in eyeblink-conditioning experiments. The standard eyeblink-conditioning paradigm was conducted in Exp. 1a (A) and Exp.1b (B), and the two-tone discrimination task of eyeblink-conditioning paradigm was conducted in Exp. 2a (C) and 2b (D).

Two types of conditioned stimuli (CS) were used, LCS (1 kHz, 80 dB, 600 ms) and HCS (10 kHz, 70 dB, 600 ms), in combination with the unconditioned stimulus (US), an electric shock (100 Hz, square pulses, 100 ms). The inter-stimulus interval of CS and US was 500 ms (Fig. 1). The US intensity was carefully determined to elicit an eyeblink and/or a head-turn and adjusted daily for each animal. Initially, two sessions of basal eyeblink responses (habituation sessions) were recorded without CS and US.

#### Experiment 1: Simple delay eyeblink conditioning

After habituation sessions, the animals were subjected to delay eyeblink conditioning for 7 days. The LCS was used in Exp. 1a (DCN lesioned mice: n = 7; sham-operated mice: n = 7) (Fig. 1A), and HCS was used in Exp. 1b (DCN lesioned mice: n = 6; sham-operated mice: n = 6) (Fig. 1B). Daily conditioning sessions consisted of 50 trials grouped in five blocks. Each block included nine CS-US paired trials and one CS-only trial. Inter-trial intervals (ITI) were pseudo-randomized between 20 s and 40 s, with a mean ITI of 30 s.

#### Experiment 2: Two-tone discrimination tasks

After habituation sessions, animals were subjected to two-tone discrimination tasks for 10 days. Daily acquisition training sessions of 50 trials were grouped into five blocks. Each block included five LCS and five HCS trials, and a total 25 LCS trials and 25 HCS trials were conducted in a daily session. In Exp. 2a (DCN-lesioned mice: n = 7; sham-operated mice: n = 8), the LCS preceded and was co-terminated with the US, including one LCS only trial in each block, and the HCS always occurred alone without the US (Fig. 1C). Each CS was presented in a pseudorandom order such that there were no more than three consecutive trials with the same CS. The same procedures used in Exp. 2a were used for Exp. 2b (DCN lesioned mice: n = 6; sham-operated mice: n = 7), except that HCS preceded and co-terminated with the US, and LCS always occurred alone and without the US (Fig. 1D). ITI were pseudo-randomized between 20 s and 40 s, with a mean ITI of 30 s.

### Data Analyses for Eyeblink Conditioning

EMG signals were band-pass filtered between 0.15 and 1.0 kHz, and fed into a computer at a sampling rate of 10 kHz. The frequency of CR occurrence was calculated from the eyelid EMG activity using the same analysis methods described by Sakamoto and Endo [16].

In Exp. 1, the threshold (mean plus SD of the EMG amplitudes) was calculated from the EMG activity amplitudes during the baseline period (300 ms before CS onset) in 50 trials. For each trial, we calculated the CR, spontaneous eyeblink responses (SER) and startle eyeblink responses (STR) based on baseline values. SER% and STR% were calculated for each session. CR% was calculated by subtracting the frequency of the SER from that of the CR in a daily 50-trial session (CR% = (CR- SER)/50×100), and was used as the learning index. We used this CR% to exclude unlearned responses (SER and responses resulting from facial and head movement) and to analyze the pure learning component, according to Sakamoto and Endo [15]. The CR% in this article is equivalent to the LER% described in Sakamoto and Endo [16]. In Exp. 2, the threshold, CR, SER, and STR were calculated in each CS trial (LCS: 25 trials, HCS: 25 trials).

Normalized EMG amplitudes were calculated from EMG amplitudes of valid trials, which were obtained by measuring the EMG 300 ms before CS onset in each mouse, and then averaging these measurements for each group of mice in Exp. 1 [11], [16]. In Exp. 2, normalized EMG amplitudes were also calculated in each CS trial (LCS: 25 trials, HCS: 25 trials).

### Rotor Rod Tests

In Exp. 2, experimental mice were subjected to the rotor rod test for 6 days before the two-tone discrimination task. The method for the rotor rod test was the same as previously described [15]. In the habituation trials before training, mice were placed on a stationary rotor rod for 5 min. In the training trials, each mouse was placed on a rotating rotor rod, and the latency to fall was recorded. The rotating speed increased gradually from 4 to 40 rpm during each training trial for a maximum trial length of 300 s. The training consisted of four trials per day for a total of 24 trials.

### Histological Methods

After behavioral experiments, mice were deeply anesthetized and transcardially perfused with phosphate-buffered saline (PBS) containing 4% paraformaldehyde. The brains were removed from the skulls, and post-fixed overnight in PBS containing 4% paraformaldehyde. Coronal sections (50 µm) were cut and stained with cresyl violet. The lesioned area was examined under a microscope.

### Statistical Analysis

Statistical analyses were performed using StatView 5.0 and Js-STAR. Two-way repeated measures ANOVA were used to analyze eyeblink conditioning and rotor-rod test data. The simple main effect test and Bonferroni test were used for *post hoc* analyses. For EMG analyses in eyeblink conditioning, the Mann-Whitney U test was used. P<0.05 was regarded as significant for all tests.

## Results

### 

#### Standard eyeblink conditioning task

In the present study, we used two types of tone-induced conditioned stimuli (LCS: 1 kHz, 80 dB and HCS: 10 kHz, 70 dB) and conducted delay eyeblink conditioning.

### Exp. 1a: DCN-lesioned Mice Acquired Conditioned Responses with the LCS

**Figure 2:**
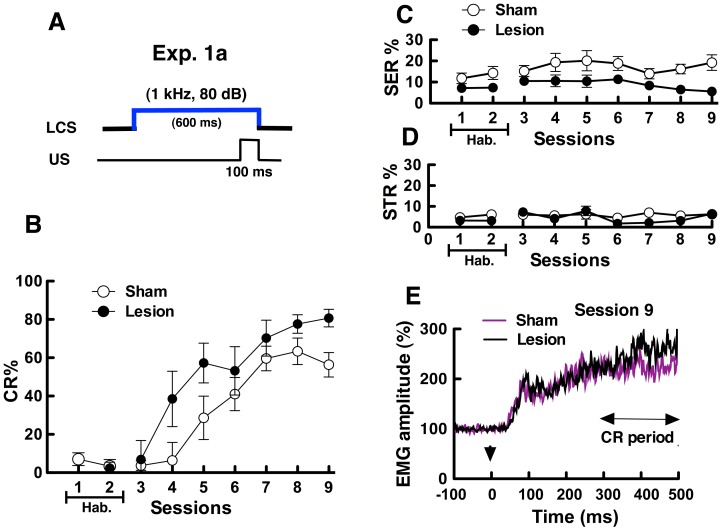
Results in simple eyeblink conditioning using low-frequency tone CS (LCS) in sham-operated and DCN-lesioned mice (Exp. 1a). (A) LCS was paired with the unconditioned stimulus (US). (B) Conditioned responses (CR)%, (C) spontaneous eyeblink responses (SER)%, and (D) startle eyeblink responses (STR)% are shown. In session 1–2, basal eyeblink responses were recorded without the CS tone and the US electric shock (Hab. means the habituation sessions.). In session 3–9, each mouse group was subjected to acquisition training. CR% was calculated by subtracting the frequency of SER from that of CR in the daily 50 trials {CR% = [(CR – SER) Ú 50]×100}. (E) Normalized EMG amplitude profiles measured during session 9. Arrowheads indicate the onset of the CS. EMG amplitudes of valid trials were normalized using the mean EMG, which was obtained by measuring the EMG 300 ms before CS onset in each mouse and then averaging these measurements for each group of mice.

In eyeblink conditioning with the LCS (Fig. 2A), both DCN-lesioned and sham-operated mice acquired the CR and learned the task (Fig. 2B). Both groups of mice showed a low CR% in the early training sessions (session 3). However, the CR% in both groups increased gradually and reached a plateau at session 9 (DCN-lesioned mice: 80.7±4.6%, sham-operated mice: 56.6±6.4%). Two-way ANOVA of the CR% with respect to group (DCN-lesioned vs. sham-operated)×session (3–9) revealed that the main effect of group (*F*_ (1,12)_ = 3.20, *P* = 0.099) and the interaction of group×session (*F*_(6,72)_ = 1.55, *P* = 0.17) was not significant, but that the main effect of the session was significant (*F*_(6,72)_ = 33.73, *P*<0.01). These results indicate that the DCN lesions did not affect the acquisition of eyeblink conditioning with LCS.

Fig. 2C and 2D show the change in SER% and STR%. Two-way ANOVA of SER% with group×session (3–9) revealed that the main effect of group was significant (*F*_(1,12)_ = 21.44, *P*<0.001), and this indicated that SER% in DCN-lesioned mice was lower than that in sham-operated mice. STR% in sham-operated and DCN-lesioned groups changed similarly, and no significant differences were observed in STR% between groups (*F*_(1,12)_ = 0.90, *P* = 0.36).

Fig. 2E shows the traces of normalized EMG amplitudes measured during valid trials in session 9. No significant differences were observed between DCN-lesioned and sham-operated mice for EMG amplitudes recorded during the CR detection periods (Mann-Whitney *U* test, *U* = 19.0, *P* = 0.48). The results show that DCN lesions did not have influence on EMG amplitudes during the CR period. Normalized EMG amplitudes in both groups increased between 50 and 100 ms from CS onset, and the short latency responses (SLRs) that are cerebellum-independent components were observed [9]. EMG amplitude in both groups increased gradually following CS onset.

### Exp. 1b: DCN-lesioned Mice Acquired Conditioned Responses with the HCS

**Figure 3:**
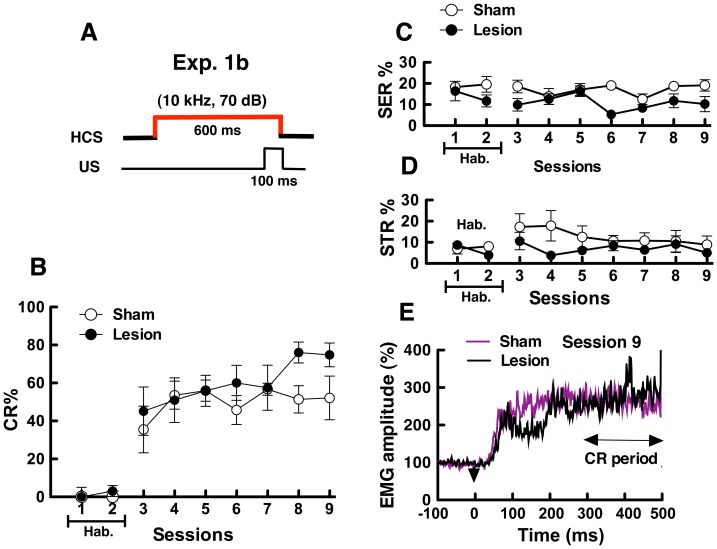
Results in simple eyeblink conditioning using high-frequency tone CS (HCS) in sham-operated and DCN-lesioned mice (Exp. 1b). (A) HCS was paired with the unconditioned stimulus (US). (B) Conditioned responses (CR)%, (C) spontaneous eyeblink responses (SER)%, and (D) startle eyeblink responses (STR)% are shown. In session 1–2, basal eyeblink responses were recorded without the CS tone and the US electric shock (Hab. means the habituation sessions.). In session 3–9, each mouse group was subjected to acquisition training. CR% was calculated as described in Fig. 2. (E) Normalized EMG amplitude profiles measured during session 9. Arrowheads indicate the onset of the CS. EMG amplitudes of valid trials were normalized as in Fig. 2.

In eyeblink conditioning with HCS (Fig. 3A), both DCN-lesioned and sham-operated mice acquired the CR (Fig. 3B). Both group mice showed some CR% (DCN mice: 45.1±12.7%, Sham mice: 35.5±12.3%) in the early training sessions (session 3). Then, CR % increased by 20–30% following the seventh training session (session 9; DCN-lesioned mice: 74.8±6.3%, sham-operated mice: 52.1±11.4%). Two-way ANOVA of the CR % with group (DCN-lesioned vs. sham-operated)×sessions (3–9) revealed that the main effect of group (*F*_ (1,10)_ = 2.15, *P* = 0.17) and session (*F*_(6,60)_ = 1.83, *P* = 0.11) as well as the interaction between group and session (*F*_(6,60)_ = 0.89, *P* = 0.51) were not significant. The results indicate that the DCN lesions did not affect the acquisition of eyeblink conditioning with HCS.

Fig. 3C and 3D show changes in SER% and STR% in each session. Two-way ANOVA on SER% with group×session (3–9) revealed that the main effect of groups was significant (*F*_(1,10)_ = 13.27, *P*<0.01), and this indicates that SER% in DCN-lesioned mice was higher than that in sham-operated mice. STR% in sham-operated and DCN-lesioned groups changed similarly, and no significant differences were observed in the STR% between groups (*F*_(1,10)_ = 1.45, *P* = 0.26).

Fig. 3E shows the traces of normalized EMG amplitudes measured during valid trials in session 9. No significant differences were observed between DCN-lesioned and sham-operated mice for EMG amplitudes recorded during the CR detection periods (Mann-Whitney *U* test, *U* = 14.0, *P* = 0.52). The results indicate that DCN lesions did not have an influence on EMG amplitudes during the CR period. Normalized EMG amplitudes in both groups increased between 50 and 100 ms from CS onset, and SLRs were clearly observed.

### Two-tone Discrimination Task

In Exp. 1, both DCN-lesioned and sham-operated mice learned standard delay eyeblink conditioning with LCS and HCS. Our previous results also suggest that the cerebellum is not so critical for mouse eyeblink conditioning when the salient CS conditions were used for conditioning [15]. We conducted two-tone discrimination tasks using the CS with two frequencies to investigate the role of DCN in higher cognitive function.

### Exp. 2a: DCN Lesions Impaired the Two-tone Discrimination Task (LCS+ vs. HCS−)

**Figure 4:**
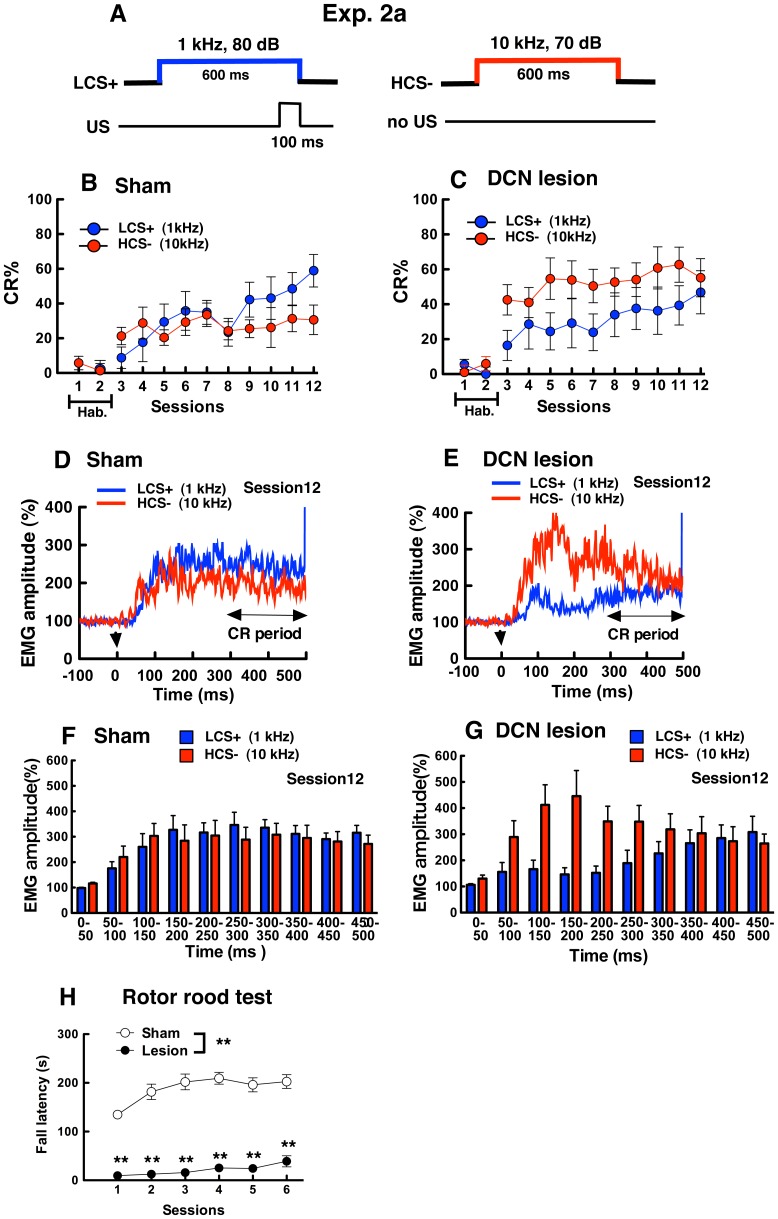
Results in the two-tone discrimination task of eyeblink conditioning and the rotor rod test in DCN-lesioned and sham-operated mice (Exp. 2a). (A) LCS+ (1 kHz) preceded and co-terminated with the US, and the HCS− (10 kHz) presented without the US. (B) (C) For eyeblink conditioning, CR% in LCS+ and HCS− trials are shown for sham-operated (B) and DCN-lesioned mice (C). In sessions 1 and 2, basal eyeblink responses were recorded without tone CS and electric shock US (Hab. means the habituation sessions.). All mice received two-tone discrimination for 10 days (sessions 3–12). CR% was calculated by subtracting the frequency of SER from those of CR in 25-LCS+ trials or 25-HCS− trials in the daily sessions {CR% = [(CR – SER) Ú 25]×100}. (D) (E) Normalized EMG amplitude profiles measured in LCS+ and HCS− trials of session 12 in sham-operated (D) and DCN-lesioned mice (E). Arrowheads indicate onset of the CS. EMG amplitudes of valid trials were normalized as in LCS+ and HCS− trials. (F) (G) Histograms of normalized EMG amplitudes in CR trials binned into 50 ms in the session 12. Normalized EMG amplitudes in CR trials were averaged in LSC+ and HSC− trials in sham-operated mice (F) and DCN lesioned mice (G). (H) The average fall latency in four trials are shown in the daily sessions in the rotor rod test. **indicates *P*<0.01 (sham-operated vs. DCN-lesion). (In the rotor rod test, sham-operated mice in session 4 were subjected to training over three trials (not four trials) owing to rotor rod device problems.).

In Exp.2a, LCS (1 kHz) was paired with the US of an electrical shock, and the HCS (10 kHz) was presented alone (Fig. 4A). In sham-operated mice, the CR% in LCS+ trials (8.8±6.1% –35.0±6.9%) was the same level as those in HCS− trials (21.3±5.0% –33.6±6.7%) from session 3 to 7. CR% in LCS+ trials (59.0±9.4%) increased gradually and was larger than those in HCS− trials (30.6±8.5%) at session 12 (Fig. 4B). Two-way ANOVA on CR% with tone frequency (LCS+ vs. HCS−) and sessions (3–12) revealed that the main effects of tone (*F*_(1,7)_ = 0.96, *P* = 0.36) were not significant, and that the main effects of session (*F*_(9,63)_ = 3.01, *P*<0.01) and interaction of tone×session (*F*_(9,63)_ = 2.03, *P* = 0.050) were significant. In the post hoc test, CR% in LCS+ trials significantly increased across the training sessions (*F*_(9,63)_ = 4.88, *P*<0.01), CR% in session 9, 10, 11, 12 was larger than that in session 3 (*P*<0.05). Furthermore, CR% in LCS+ trials was larger than that in HCS− at session 9, 10, 11, 12 although they are not statistically significant. These results suggest that sham-operated mice discriminated the LCS+ and HCS− to acquire the CR.

In contrast, in DCN-lesioned mice, CR% in LCS+ trials (16.4±8.6% –46.8±12.3%) were smaller than those in HCS− trials (42.5±8.7% –52.2±10.9%) throughout the sessions (Fig. 4C). Two-way ANOVA on CR% revealed that the main effects of tone frequency (*F*_(1,6)_ = 2.07, *P* = 0.20), sessions (*F*_(9,54)_ = 1.90, *P* = 0.072) and interaction of tone×sessions (*F*_(9,54)_ = 0.39, *P* = 0.93) were not significant. The results indicate that DCN-lesioned mice did not discriminate between LCS+ and HCS−. DCN-lesioned mice seemed to express eyeblink responses regardless of CS frequency (with or without the US).

Fig. 4D and 4E shows the normalized EMG amplitudes measured during valid trials in session 12. EMG amplitudes in the CR detection period of LCS+ trials and HCS− trials were compared in each group. In sham-operated mice, EMG amplitudes in LCS+ trials were larger than those in HCS− trials, although statistically significant differences were not observed (Fig. 4D: Mann-Whitney *U* test, *U* = 16.0, *P* = 0.093). In DCN-lesioned mice, no significant differences were observed in EMG amplitude between LCS+ and HCS− trials at the CR detection period (Fig. 4E: *U* = 20.0, *P* = 0.57). EMG amplitude also indicated that DCN-lesioned mice did not discriminate between LCS+ and HCS−.

To examine the onset and peak latency of CR in LCS+ trials and HCS− trials, we analyzed normalized EMG amplitude for 500 ms from CS onset to US onset. Fig. 4F and Fig. 4G show histograms of normalized EMG amplitudes in CR trials binned into 50 ms in the session 12. In both sham-operated and DCN lesioned mice, the onset latency was observed at early time ranges (50–100 ms) in LCS+ and HCS− trails. The peak EMG amplitude in HCS− trials in DCN lesioned mice was larger than that in sham-operated mice. In the LSC+ trials, the peak latency in DCN lesioned mice (400–500 ms) appeared late compared with sham-operated mice (150–250 ms).

Rotor rod tests were conducted before eyeblink conditioning experiments to examine motor learning and coordination. Fig. 4H shows the averaged fall latency of four trials in sham-operated and DCN-lesioned mice. Sham-operated mice learned this task and their fall latency increased across the sessions (sessions 1: 134.8±5.6 s, sessions 6: 202.7±14.6 s). In contrast, DCN-lesioned mice showed severe impairment and fall latency was smaller than that in sham-operated mice (sessions 1: 9.5±2.16 s, sessions 6: 39.0±11.44 s). Two-way ANOVA on fall latency within groups (sham-operated vs. DCN-lesion) and sessions (1–6) revealed that the main effects of groups (*F*_ (1,13)_ = 167.85, *P*<0.01) and sessions (*F*_ (5,65)_ = 12.40, *P*<0.01) as well as the interaction of groups×sessions (*F*_ (5,65)_ = 4.74, *P*<0.01) were significant. In the *post hoc* test, fall latency in DCN-lesioned mice was smaller than that in sham-operated mice at all sessions (*Ps* <0.01).

### DCN Lesions Impaired the Two-tone Discrimination Task (HCS+ vs. LCS−)

**Figure 5:**
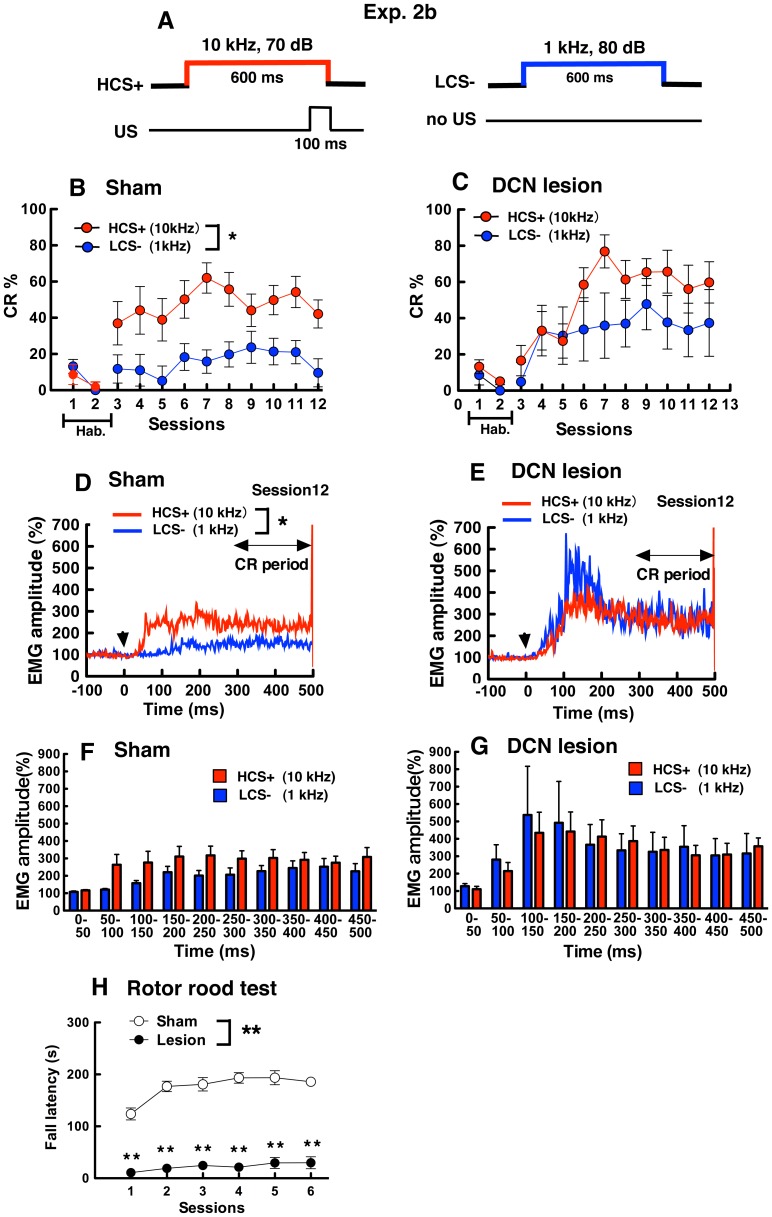
Results in the two-tone discrimination task of eyeblink conditioning and the rotor rod test in DCN-lesioned and sham-operated mice (Exp. 2b). (A) HCS+ (10 kHz) preceded and co-terminated with the US, and the LCS− (1 kHz) was presented without the US. (B) (C) Results of CR% in HCS+ and LCS− trials are shown for sham-operated (B) and DCN-lesioned mice (C). In sessions 1 and 2, basal eyeblink responses were recorded without tone CS and electric shock US (Hab. means the habituation sessions.). All mice received two-tone discrimination task for 10 days (sessions 3–12). CR% was calculated as described in Fig. 4. (D) (E) Normalized EMG amplitude profiles measured in HCS+ and LCS− trials of session 12 in sham-operated (D) and DCN-lesioned mice (E). Arrowheads indicate the onset of the CS. EMG amplitudes of valid trials were normalized in HCS+ and LCS− trials. (F) (G) Histograms of normalized EMG amplitudes in CR trials binned into 50 ms in the session 12. Normalized EMG amplitudes in CR trials were averaged in HSC+ and LSC− trials in sham-operated mice (F) and DCN lesioned mice (G). (H) Averaged fall latencies in four trials are shown for the daily sessions. *indicates *P*<0.05 (HCS+ vs. LCS−); **indicates *P*<0.01 (sham-operated vs. DCN-lesioned).

In Exp. 2b, HCS (10 kHz) was paired with electrical shock (US), and LCS (1 kHz) occurred alone (Fig. 5A). Sham-operated mice showed high CR% (36.9±12.0% –62.0±8.4) in HCS+ trials, but low CR% (5.2±8.2% –23.7±8.8) in LCS− trials across the training sessions (Fig. 5B). In the sham-operated mice, two-way ANOVA on CR% with tone frequency (HCS+ vs. LCS−) and sessions (3–12) revealed that the main effects of tone were significant (*F*_ (1,6)_ = 15.34, *P*<0.01); however, the main effects of sessions (*F*_(9,54)_ = 1.74, *P* = 0.10) and interaction of tone×sessions (*F*_(9,54)_ = 0.54, *P* = 0.84) were not significant. These results indicate that sham-operated mice could discriminate between HCS+ and LCS− to acquire the CR.

In contrast, CR% in DCN-lesioned mice increased in both HCS+ trials (16.6±8.4% –65.7±11.8) and LCS− trials (4.8±10.7% –47.8±14.1) throughout the sessions (Fig. 5C). In DCN-lesioned mice, two-way ANOVA on CR% with tone frequency×sessions revealed that the main effects of tone (*F*_ (1,5)_ = 2.27, *P* = 0.19) and the interaction of tone×sessions were not significant (*F*_ (9, 45)_ = 1.77, *P* = 0.10), and the main effect of sessions was significant (*F*_ (9,45)_ = 3.16, *P*<0.01). These results indicate that DCN-lesioned mice did not discriminate between HCS+ and LCS−. Individual CR% in DCN lesion ned mice were examined, because the error bars in the LCS− trails were relatively large in Exp 2b. Two DCN lesioned mice showed high CR% (57–100%) at session 4 to10 in the LCS− trials (Fig. 5C).

Fig. 5D and 5E show the normalized EMG amplitudes measured during valid trials in session 12. EMG amplitudes in the CR detection period of HCS+ trials and LCS− trials were compared in each group. In sham-operated mice, EMG amplitudes in HCS+ trials were larger than that in LCS− trials (Fig. 5D: Mann-Whitney *U* test, *U* = 8.0, *P*<0.05); however, no significant differences were observed in EMG amplitudes between HCS+ and LCS− trials in DCN-lesioned mice (Fig. 5E: *U* = 13.0, *P* = 0.42). These results in EMG amplitude also indicate that sham-operated mice discriminated between HCS+ and LCS−, but not DCN-lesioned mice.

To examine the onset and peak latency of CR in HCS+ trials and LCS− trials, we analyzed normalized EMG amplitude for 500 ms from CS onset to US onset. Fig. 5F and Fig. 5G show histograms of normalized EMG amplitudes in CR trials binned into 50 ms in the session 12. In both sham-operated and DCN lesioned mice, the onset latency was observed at early time ranges (50–150 ms) in HCS+ and LCS− trails. The peak EMG amplitude in HCS+ and LCS− trials in DCN-lesioned mice was larger than that in sham-operated mice.

In the rotor rod tests (Fig. 5H), sham-operated mice learned this task and their fall latency increased across sessions (sessions 1: 123.9±11.6 s, sessions 6: 185.9±7.0 s). In contrast, DCN-lesioned mice showed severe impairment and their fall latency (sessions 1: 10.8±5.1 s, sessions 6: 29.9±11.4 s) was smaller than that in sham-operated mice. Two-way ANOVA on fall latency within groups (sham-operated vs. DCN-lesion) and sessions (1–6) revealed that the main effects of groups (*F*_ (1,11)_ = 169.30, *P*<0.01) and sessions (*F*_ (5,55)_ = 12.77, *P*<0.01), and the interaction of groups×sessions (*F*_ (5,55)_ = 5.09, *P*<0.01) were significant. In the *post hoc* test, fall latency in DCN-lesioned mice was smaller than that in sham-operated mice at all sessions (*Ps* <0.01).

### Identification of Lesions in DCN-lesioned Mice

**Figure 6:**
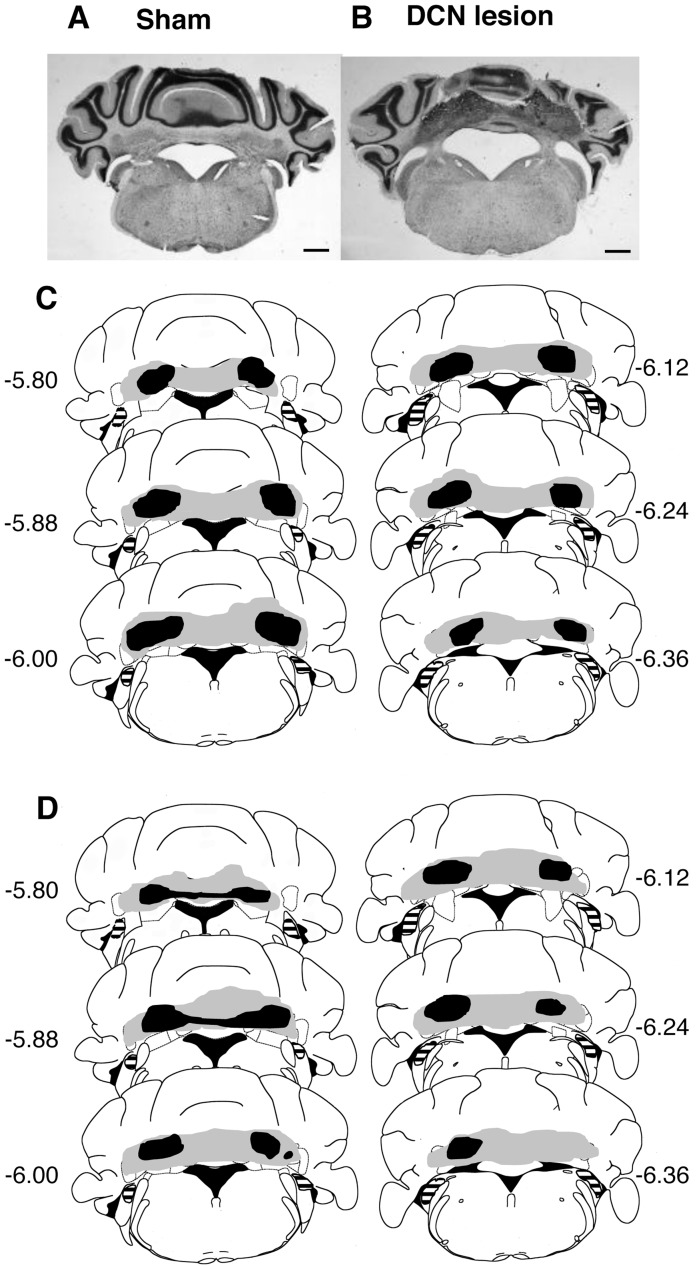
Identification of deep cerebellar nuclei (DCN) lesions. (A) (B) Representative cresyl violet-stained brain sections (50 µm) in sham-operated (A) and DCN-lesioned mice (B). (C) (D) The black and gray shaded areas represent the smallest and the most extensive lesions, respectively in Experiment 1 (C) and Experiment 2 (D). The horizontal-striped areas indicate the dorsal cochlea nucleus. Numbers represent the anteroposterior distance (mm) of the sections relative to bregma. Scale bar = 1 mm.

DCN lesions were verified after all behavioral tests according to Sakamoto and Endo [15]. The DCN was divided into five subregions: anterior interpositus nucleus, posterior interpositus nucleus, lateral nucleus, anterior medial nucleus, and posterior medial nucleus [27]. We excluded animals from analysis when the anterior interpositus nucleus was intact or when more than 50% of the DCN was intact. On average, 88.9% of the DCN was lesioned, including the entire anterior interpositus nucleus. Figure 6A and 6B show representative cresyl violet-stained brain sections in sham-operated (Fig. 6A) and DCN-lesioned mice (Fig. 6B). Figure 6C and 6D show smallest (Black) and the most extensive (gray) lesions, respectively in Expriment 1 (6C) and Expriment 2 (6D). In all DCN-lesioned mice, dorsal cochlea nuclei were intact (Fig. 6C, 6D). Performances of DCN lesioned mice did not relate the lesion size and placement among mice in both Exp.1 and Exp.2.

## Discussion

In our previous studies, both DCN-lesioned and sham-operated mice acquired the CR in delay eyeblink conditioning under salient CS conditions (10 kHz, 70dB) [15]. These results indicated that the cerebellum is not critical for simple eyeblink conditioning when the salient CS is used. Recently, the cerebellum has been reported to be involved in not only motor learning but also high cognitive functions such as spatial memory, working memory, and visual discrimination in rodents, monkeys, and humans [17], [19], [20], [21], [24], [28]. Therefore, we hypothesized that the cerebellum plays a role in more complicated associative learning, and investigated its role in the two-tone discrimination task in the eyeblink-conditioning paradigm using DCN-lesioned mice.

In Exp. 1, DCN-lesioned and sham-operated mice were subjected to simple eyeblink conditioning under a LCS or HCS. Both sham-operated and DCN-lesioned mice acquired the CR in eyeblink conditioning under LCS (1 kHz, 80dB) conditions (Fig. 2B) and HCS (10 kHz, 70dB) conditions (Fig. 3B). The results indicate that DCN is not so critical for standard eyeblink conditioning. We revealed that the inactivation of the cerebellum impaired eyeblink conditioning when the CS of a lower decibel was used (10 kHz: 61dB; [16]). Taken together, these findings suggest that the DCN is not critical for the acquisition of CR in eyeblink conditioning under salient CS conditions, such as 10 kHz-70 dB and 1 kHz-80 dB, but not 10 kHz-61 dB [15], [16]. In both CS conditions, SLR were observed in EMG amplitudes (Fig. 2E and Fig. 3E), which is independent of the cerebellum [9]. In previous works, SLR were observed also in rabbits with lesions in the cerebellar cortex [29], [30]. These findings suggest that SLR are induced through the extracerebellar pathway. In the mice, lesions of the amygdala impaired CR and SLR in mice [15], and suggest that SLR induction requires the involvement of in forebrain system including the amygdala.

DCN-lesioned mice were subjected to two-tone discrimination tasks in Exp. 2. In Exp. 2a, 1 kHz CS (LCS+) was paired with the US but 10 kHz was not (HCS−) during eyeblink conditioning (Fig. 4A). CR% in sham-operated mice gradually increased, indicating that mice learned the two-tone discrimination task (Fig. 4B). In contrast, DCN-lesioned mice showed higher CR% in the HCS− trials than in the LCS+ trials throughout all sessions, indicating they could not learn the two-tone discrimination task (Fig. 4C). In Exp. 2b, CS tones were switched; 10 kHz CS (HCS+) was paired with the US but 1 kHz was not paired (LCS−) during eyeblink conditioning. The sham-operated mice showed high CR% in the HCS+ trials and low CR% in the LCS− trials (Fig. 5B). DCN-lesioned mice showed high CR% not only in HCS+ trials but also in LCS− trials at session 12 (Fig. 5C). These results also indicated that DCN lesions impaired the two-tone discrimination task in the eyeblink-conditioning paradigm.

According to behavioral audiograms in the C57BL/6J mouse, optimal tone frequencies for the normal mouse are 8–32 kHz [31]. Consistent with the findings, in the present study, sham-operated mice learned faster 10 kHz CS (Fig. 2B) than 1 kHz CS (Fig. 2A) in simple eyeblink conditioning (Exp.1). In addition, the conditioning for LCS+ (Fig. 4B) seems to be more difficult compared with that for HCS+ (Fig. 5B) in the discrimination task (Exp.2). This tendency that responded strongly to HCS tone than LCS tone in sham-operated mice was observed even after DCN lesions (Fig. 4C and Fig. 5C). In Exp 2, DCN lesioned mice showed high CR% in the CS- trials (Fig. 4C, 5C), in particular in the HCS− trials (Fig. 4C). These results suggest that DCN lesions might induce sensitization in the CS- trials by effects of CS+ trials, or they might facilitate generalization in the CS- trials as the result of association of CS and US in CS+ trials. In the analyses of normalized EMG trace in CR trials, the peak EMG amplitude in HCS− trials (Fig. 4F and 4G) and LCS− trials (Fig. 5F and 5G) in DCN lesioned mice appeared to be larger than that in sham-operated mice. The results indicate that DCN lesions may have enhanced the sensitivity to the HCS and LCS in the CS- trials. To reveal the role of the cerebellum in two-tone discrimination learning, in the further experiments, examination of the relationship between the cerebellum and the sensitivity of the tone CS will be required.

We found for the first time that the cerebellum is critical for the discrimination of tone frequency in eyeblink-conditioning paradigms. We revealed that in mice the amygdala plays an important role in eyeblink conditioning but not the cerebellum under salient CS [15]. The present study again indicates that DCN is not necessary for standard delay eyeblink conditioning under salient CS. The two-tone discrimination task has two aspects, the acquisition of CR to one CS and the inhibition of CR to the other CS [32], and is required more complicated learning processes compared to simple eyeblink conditioning. Our findings indicate that DCN plays important roles in more complicated associative learning, such as the discrimination of tone frequency in eyeblink conditioning paradigms.

The two-tone discrimination task and the reversal task have been conducted to examine the cognitive function in the hippocampus [33], [34] or the cerebellum [35] in rabbits. Hippocampal lesioned rabbits showed impairment in the reversal discrimination task, but not initial discrimination task [33]. In the unit recording study, the CR related activity in the hippocampus was observed during CS+ trials in both the discrimination and the reversal tasks [34]. In contrast, the CR related activity in the DCN (interpositus nucleus) was specifically observed during the CS+ trials in the discrimination task, but not the reversal task [35]. These results support our findings that DCN was involved in the two-tone discrimination task. DCN-lesioned rats showed impairment in cognitive processes in the supra-second temporal discrimination task [36]. This finding suggests that the cerebellum may be involved in not only the discrimination of tone frequency but also in timing discrimination of the CS.

We demonstrated that DCN plays an important role in the two-tone discrimination task (Fig. 4B, 4C, Fig. 5B, 5C), then, what are the underlying neural mechanisms? The cerebellar cortex may play an important role in this task, because DCN lesions impair not only physiological functions of the DCN but disturb the flow of information from the cerebellar cortex to downstream neural pathway. For example, neural activity in the cerebellar cortex was induced by passive listening and modulated by frequency (pitch) discrimination task in human [37]. The subcortical short loop between cerebellum and the olivochochlear system is another candidate for the fine control of auditory stimulus [37], [38]. The cerebellum sends direct efferent projections to divisions of the inferior colliculus, which conveys sound frequency information [39]. In mice, on the other hand, involvement of auditory cortex is reported in this learning [40]–[42], however, there is no report of the cerebellar involvement in this task.

Recently, the cerebellum has been reported to be critical not only for motor coordination and learning but also for cognitive function in animals, including humans [17], [21], [43]. Cerebellar mutant mice such as Purkinje cell degeneration (*pcd*) mutant mice [28] and *nervous* mutant mice [20] showed impairment in the place task water maze but not the cue task water maze, and cerebellum lesions caused severe defects in the right/left discrimination task in rats [24]. In humans, marked activation of the cerebellum was reported during mental recall of personal episodes [44] and fear conditioning [45] in normal subjects. These behavioral and physiological findings are supported by anatomical studies, in which the prefrontal cortex and motor cortex connected to the cerebellum via the pontine nuclei and the thalamus [17], [46]–[48].

In summary, we revealed that DCN play an important role in the discrimination of tone frequency in eyeblink conditioning paradigms. This finding suggests that the cerebellum is involved in complicated associative learning. The role of the cerebellum on other psychological functions, for example, socio-emotional function such as emotionality, communication, and social interaction, need to also be examined in the future.
